# Secondary IOL Implantation without Capsular Support: A Laser Flare Cell Meter Study

**DOI:** 10.5402/2011/653246

**Published:** 2012-01-02

**Authors:** Mauro Cellini, Ernesto Strobbe, Pier Giorgio Toschi, Emilio C. Campos

**Affiliations:** Department of Surgery Science and Anesthesiology, Ophthalmology Service, University of Bologna, Via Palagi 9, 40138 Bologna, Italy

## Abstract

Phacoemulsification and the contemporary implantation of intraocular lens (IOL) within the capsular bag represent the standard of care in cataract surgery, but sometimes a primary IOL implant is not possible due to intraoperative complications or preexisting conditions so that a secondary implantation of IOL within the anterior or posterior chamber is necessary. The aim of our study was to assess the degree of inflammation due to a secondary implant of claw lenses, angle-supported IOLs, and scleral-fixated IOLs by means of an objective, repeatable, and noninvasive device, the laser flare cell meter, which evaluates aqueous flare and cells within the anterior chamber in vivo and to show the contribution of the single IOLs to the genesis of inflammation.

## 1. Introduction

Modern cataract surgery involves phacoemulsification of the opacified crystalline lens and the implant of an intraocular lens (IOL) in the capsular bag. In some conditions this is not possible due to the type of cataract (e.g., traumatic cataracts with lens subluxation, cataracts in pseudoexfoliative syndrome with zonular/capsular dehiscence) or to systemic and congenital disorders characterized by weakness of zonules/capsule (e.g., familial or idiopathic ectopia lentis, Marfan Syndrome, etc.) or to intraoperative complications (e.g., large breaks of the posterior capsule, accidental aspiration of the capsular bag, etc.).

In these cases it is necessary to perform a secondary implant which may be a scleral-fixated posterior chamber IOL (SPCIOLs), an angle-supported anterior chamber IOL (AACIOLs), or an iris-fixated anterior chamber IOL (IACIOLs) [1]. Moreover, the use of these types of IOLs and the stimulation of the irideal tissue and the ciliary bodies may cause the onset of an inflammatory reaction that can manifest itself as uveitis, but in most cases remains subclinical [2, 3].

In clinical practice, slit-lamp biomicroscopy has been the method used to assess the degree of inflammation, aqueous flare, and cells within the anterior chamber; however this method is only qualitative and subjective. Several attempts have been made to develop instruments to quantify aqueous flare intensity [4–6]. Fluorophotometry is a quantitative method that evaluates the permeability of the blood-aqueous barrier. Nevertheless the need for fluorescein injection, the duration of the test, and the possible adverse effects related to the dye limit the clinical applications of this technique [7].

Moreover another method may be used for a quantitative clinical assessment of intraocular inflammation: the laser cell flare meter which determines protein concentration and cell number in aqueous humor in vivo [8].

The purpose of this study was to assess the presence of any subclinical chronic inflammation following secondary implantation of IACIOLs, SPCIOLs, and AACIOLs using the laser cell flare meter.

## 2. Materials and Methods

A total of 60 patients were enrolled, 34 males and 26 females, aged between 26 and 88 years (mean 60 ± 8.3), aphakic and without capsular support, for a total of 60 eyes. Absence of capsular support in 20 patients was due to a previous extracapsular extraction of a traumatic cataract following a penetrating bulbar wound, in 26 to a large break in the posterior lens capsule during phacoemulsification of senile hard cataracts, and in 14 to a subluxation of the capsular bag following phacoemulsification.

Eligible patients were randomly divided into three groups: the first group (A) was implanted with an iris-fixated IOL (Artisan-Ophtec BV, Groningen, Netherlands), the second group (B) with a scleral-fixated posterior chamber IOL (PC 279Y-Ophtec BV, Groningen, Netherlands), and the third group (C) with an angle-supported IOL model “Kelman” (Surgidev Inc., N.J., USA).

All patients were operated by the same surgeon in the S. Orsola-Malpighi Hospital Ophthalmology Service.

Approval was obtained from the institutional S. Orsola-Malpighi Hospital Ethics Committee. Before participating in the study, all patients provided signed informed consent after a detailed description of the surgical procedures and an accurate explanation of the aim of the study by the surgeon.

Each patient underwent the following preoperative examinations: measurement of visual acuity, applanation tonometry, anterior and posterior segment biomicroscopy, iridocorneal angle evaluation, specular microscopy, assessment of pupil motility, and echobiometry for calculation of the IOL power.

Exclusion criteria were as follows:

- patients with ocular hypertension exceeding 24 mmHg, not controlled by medical treatment,
- patients with macular degeneration,
- patients with recurrent uveitis,
- patients undergoing other types of eye surgery,
- patients with an endothelial cell count of less than 1500 cell/mm^2^.

Surrounding adnexae were cleaned with a 10% povidone-iodine solution and the eye with a 5% povidone-iodine solution for 3 minutes and then washed away by balanced saline solution.

For the implant of IACIOLs, two paracentesis at 2.30 and 9.30 were made. A sclerocorneal incision of 5.4 mm at 12 o'clock was made and anterior vitrectomy performed if necessary.

Acetylcholine (Miochol, Novartis Ophthalmics, Basel, Switzerland) was injected to constrict the pupil followed by a high molecular weight viscoelastic substance. The IOL was inserted along its minor diameter using the appropriate Verisyse gripper. Once within the anterior chamber, the lens was turned by 90° and positioned using the appropriate Verisyse lens manipulator. Then the midperipheral iris was grasped and pulled through the claws at the 3 and 9 o'clock positions, and a peripheral iridectomy was performed. At the end of the surgery viscoelastic was removed and replaced with a balanced saline solution (BSS) to ensure good anterior chamber depth, and the incision was sutured with single nylon 10-0 sutures.

The SPCIOL's implantation technique included, before surgery, the administration of cyclopentolate 1% eye drops. A 180° superior conjunctival peritomy was performed. Two triangular partial thickness, 3 × 3 mm, scleral flaps were fashionated at 3 and 9 o'clock. Removal of any vitreous in the anterior chamber was performed. A high weight viscoelastic agent was injected into the anterior chamber. Then a 7 mm corneal incision was made at 12 o'clock for the insertion of the suture needles and the PCIOL. Two Kelman needles attached to a 10.0 polypropylene suture were passed around the distal portion or tip of the IOL loop, were inserted through the main corneal tunnel, passed behind the iris, and extracted through the bed of the two scleral flaps 1,5 mm posterior to the limbus.

Subsequently the IOL was inserted within the posterior chamber. Then the two sutures were gently tightened to secure the final position of the IOL behind the iris. Viscoelastic was removed, and finally the scleral flaps and the corneal wound were closed with 10.0 nylon sutures and the conjunctiva with vicryl 8-0 sutures.

For AACIOL's implantation, a temporal paracentesis was made to inject acetylcholine (Miochol, Novartis Ophthalmics, Basel, Switzerland) followed by a high molecular weight viscoelastic into the anterior chamber.

Where necessary, an anterior mechanical vitrectomy was performed to remove any vitreous in the anterior chamber.

Then a 7 mm incision was made 1-2 mm posterior to the superior limbus, and the IOL was inserted with the help of a glide, taking care to avoid catching iris tissue. Its distal haptic was positioned in the inferior angle; then the proximal haptic was positioned in the superior angle of the anterior chamber. A peripheral iridectomy was performed. Viscoelastic was removed, and the scleral wound was sutured closed with single 10.0 nylon sutures.

The control group included 18 patients, 10 males and 8 females, aged between 63 and 78 years (mean 71.4 ± 4.5), for a total of 18 eyes undergoing phacoemulsification and IOL implantation in the capsular bag (PCIOL's).

To assess postsurgical inflammatory reaction, 30 and 90 days after surgery, patients were examined with a laser cell flare meter FC-2000 (Kowa Company, Ltd., Electronics and Optics Division, Tokjo, Japan) which quantifies aqueous humor proteins and cells.

The laser flare cell meter consists of an He-Ne laser beam system, a photomultiplier mounted on a slit-lamp biomicroscope, and a computer. The laser scans the aqueous humor across a sampling window (0.3 × 0.5 mm) over 0.5 sec by means of an optical scanner. Light scattered by protein particles and inflammatory cells in the aqueous is proportional to their concentration and size and is detected by a photon-counting multiplier and processed by a computer. Given that cells are larger than proteins, the amount of light scattered by cells is greater than that reflected by fine protein particles.

At the end of the measurement flare is expressed in photon counts per milliseconds (ph/msec).

A total of 7 measurements were obtained for each eye, the highest and the lowest values were eliminated, and the mean and standard deviation were calculated automatically by the computer and then analysed statistically. Statistical analysis was performed with the Wilcoxon signed-rank test considering *P* < 0.05 as positive.

## 3. Results

**Figure 1:**
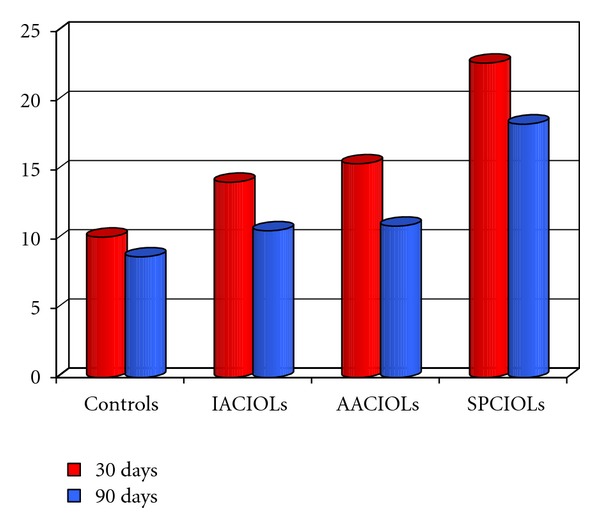
Flare values 30 and 90 days after surgery in the IACIOLs (iris-fixated anterior-chamber IOL), AACIOLs (angle-supported anterior chamber IOL), SPCIOLs (scleral-fixated posterior chamber IOL), and Controls.

As shown in Tables 1, 2, and 3 and in Figure 1, there was an increase in flare values of 52,5% (*P* < 0,018) after 30 days and 25,3% (*P* < 0,034) after 90 days in the group C, of 124,7% (*P* < 0,001) after 30 days and 110,3% (*P* < 0,001) after 90 days in the group B, and of 39,6% (*P* < 0,021) after 30 days and 21,8% (*P* < 0,032) after 90 days in the group A, compared with the control group.

The SPCIOL's group showed an increase in the flare value of 47,4% (*P* < 0,004) after 30 days and 67,8% (*P* < 0,003) after 90 days compared with the group C whereas the increase was 60,9% (*P* < 0,001) after 30 days and 72,6% (*P* < 0,001) after 90 days compared with the group A. Finally, the AACIOL's group showed an increase in flare of 9,2% (*P* < 0,376) after 30 days and 2,8% (*P* < 0,507) after 90 days compared with the IACIOL's group but it was not statistically significant.

## 4. Discussion

IOL implants in cataract surgery can be primary or secondary. The primary implants are performed always after a noncomplicated cataract surgery, whereas the secondary implants tend to be performed after the cataract removal, even months or years later. Several conditions can prevent a primary implant. They are usually intraoperative complications, but also trauma, crystalline ectopia, crystalline subluxation, weakness of the zonules, or previous lensectomy during childhood are conditions in which secondary implants are necessary.

The main secondary IOL implants actually used are scleral-fixated, angle-supported, and iris-fixated IOLs [1].

To date, in the few trials performed to compare the different types of lenses or in the studies on the individual IOLs, there is insufficient evidence to demonstrate the clear superiority of one lens type or fixation site [9–15]. The results of IOL implantation in the absence of capsule support have been satisfactory in general when viewed with respect to visual results and recovery [9–11, 15–20], although few trials appear to show that anterior chamber IOLs provide a superior visual recovery than scleral-fixated PCIOLs [21–23]. The cause for the poorer visual recovery with sclera-fixated PCIOLs is represented by microscope light-induced retinal injuries, due to the prolonged operating time compared with anterior chamber implants [12, 24]. This is confirmed by an angiographic study that showed how the incidence of retinal damage caused by phototrauma in eyes undergoing scleral-fixated PCIOLs was 33% [25].

As regards the onset of a more or less marked postsurgical inflammatory reaction, the data in literature are very heterogeneous because they cover different surgical techniques and several types of lenses [26–29]. Some studies have however demonstrated how the onset of cystoid macular edema varies from 6.7% to 42.9% following scleral-fixated IOL implants while it is drastically reduced from 5.7% to 22.2% with anterior chamber IOL implants [11, 19, 30–35]. Thus the laser flare meter data we collected in this study, highlight that, following secondary IOL implantation, due to the absence of capsular support, there is a subclinical inflammation that is still present three months after surgery and is statistically significant when compared with flare values of patients undergoing phacoemulsification and contemporary IOL implantation in the capsular bag. Anterior chamber IOLs are responsible of a minor subclinical intraocular inflammatory reaction compared with scleral-fixated posterior chamber IOLs.

In fact we believe that this is due to some disadvantages of sclera-fixated IOLs, such as the greater technical complexity, increased operating time, and surgical manipulations in the region of the ciliary body which may cause a greater risk of damaging vascular uveal tissue with consequent cyclitic reactions, hyphema, and vitreous and suprachoroidal hemorrhages [36–40].

Moreover there is no statistically significant difference in the flare values between the two anterior chamber intraocular lenses we used to correct aphakia although there is less inflammatory reaction in the aqueous humor of the iris-fixated IOLs.

## 5. Conclusion

From our study we can confirm that iris-fixated anterior chamber IOLs and angle-supported anterior chamber IOLs cause a lower incidence of chronic subclinical inflammatory reaction than scleral-fixated posterior chamber IOLs at 30 and 90 days after surgery. Thus we suggest their implant for the correction of aphakia in the absence of capsular support not only because they cause a reduced inflammation but also because surgical technique is simpler and faster.

## Figures and Tables

**Table 1:** Flare values 30 and 90 days after surgery in Group A (IACIOLs), in Group B (SPCIOLs), in Group C (AACIOLs), and Controls.

	Controls (ph/msec)	Group A (ph/msec)	*P* < 0.05	Group B (ph/msec)	*P* < 0.05*	Group C (ph/msec)	*P* < 0.05**
30 days	10.14 ± 3.43	14.11 ± 7.09	0.021	22.72 ± 6.63	0.001	15.44 ± 10.43	0.018
90 days	8.72 ± 2.73	10.60 ± 3.96	0.032	18.31 ± 6.03	0.001	10.94 ± 7.39	0.034

*Statistical analysis between Group B and Controls.

**Statistical analysis between Group C and Controls.

**Table 2:** Flare values 30 and 90 days after surgery in Group A and Group C.

	Group A (ph/msec)	Group C (ph/msec)	*P* < 0.05
30 days	14.11 ± 7.09	15.44 ± 10.43	0.376
90 days	10.60 ± 3.96	10.94 ± 7.39	0.507

**Table 3:** Flare values 30 and 90 days after surgery in Group A, in Group B, and in Group C.

	Group A (ph/msec)	Group B (ph/msec)	*P* < 0.05	Group C (ph/msec)	*P* < 0.05°
30 days	14.11 ± 7.09	22.72 ± 6.63	0.001	15.44 ± 10.43	0.004
90 days	10.60 ± 3.96	18.31 ± 6.03	0.001	10.94 ± 7.39	0.003

°Statistical analysis between Group B and Group C.
